# The Molecular Basis of Heat-Stable Enterotoxin for Vaccine Development and Cancer Cell Detection

**DOI:** 10.3390/molecules28031128

**Published:** 2023-01-23

**Authors:** Masaya Goto, Shinya Yoshino, Kyona Hiroshima, Toru Kawakami, Kaeko Murota, Shigeru Shimamoto, Yuji Hidaka

**Affiliations:** 1 Faculty of Science and Engineering, Kindai University, 3-4-1 Kowakae, Higashi-Osaka, Osaka 577-8502, Japan; 2 Institute for Protein Research, Osaka University, 3-2 Yamadaoka, Suita, Osaka 565-0871, Japan; 3 Faculty of Life and Environmental Sciences, Shimane University, 1060 Nishikawatsu, Matsue, Shimane 690-8504, Japan

**Keywords:** cancer, disulfide, guanylyl cyclase, toxoid, vaccine

## Abstract

Heat-stable enterotoxin (ST_a_) produced by Enterotoxigenic *E. coli* is responsible for causing acute diarrhea in infants in developing countries. However, the chemical synthesis of ST_a_ peptides with the native conformation and the correct intra-molecular disulfide bonds is a major hurdle for vaccine development. To address this issue, we herein report on the design and preparation of ST_a_ analogs and a convenient chemical method for obtaining ST_a_ molecules with the correct conformation. To develop an ST_a_ vaccine, we focused on a structure in a type II β-turn in the ST_a_ molecule and introduced a D-Lys residue as a conjugation site for carrier proteins. In addition, the -Glu-Leu- sequence in the ST_a_ molecule was replaced with a -Asp-Val- sequence to decrease the toxic activity of the peptide to make it more amenable for use in vaccinations. To solve several issues associated with the synthesis of ST_a_, such as the formation of non-native disulfide isomers, the native disulfide pairings were regioselectively formed in a stepwise manner. A native form or topological isomer of the designed ST_a_ peptide, which possesses a right-handed or a left-handed spiral structure, respectively, were synthesized in high synthetic yields. The conformation of the synthetic ST_a_ peptide was also confirmed by CD and NMR spectroscopy. To further utilize the designed ST_a_ peptide, it was labeled with fluorescein for fluorescent detection, since recent studies have also focused on the use of ST_a_ for detecting cancer cells, such as Caco-2 and T84. The labeled ST_a_ peptide was able to specifically and efficiently detect 293T cells expressing the recombinant ST_a_ receptor (GC-C) protein and Caco-2 cells. The findings reported here provide an outline of the molecular basis for using ST_a_ for vaccine development and in the detection of cancer cells.

## 1. Introduction

Enterotoxigenic *Escherichia coli* (ETEC) is responsible for causing approximately 380,000 deaths annually by producing heat-stable enterotoxin (ST_a_) alone or with heat-labile enterotoxin (LT) [1]. ST_a_ stimulates the action of guanylyl cyclase C (GC-C), resulting in an increase in the concentration of intracellular cGMP levels. This activates several effectors, including the cGMP-dependent protein kinase, leading to the phosphorylation of the cystic fibrosis transmembrane conductance regulator (CFTR) and ultimately causes acute diarrhea in infants [2,3]. GC-C is a membrane-bound protein that functions as a receptor for peptide hormones, guanylin and uroguanylin, and is expressed in the intestine, but also in the kidney and brain [2,3,4,5]. It is noteworthy that GC-C is expressed at relatively high levels in cancer cells, such as Caco-2 and T84 cells [6,7]. ST_a_ is, therefore, a potential target peptide in terms of preparing an ETEC vaccine and for the detection of cancer cells. However, the characteristic structure of the ST_a_ molecule, in which the native pairings of intra-molecular disulfide bonds are required for its toxicity, has been a road block to the chemical syntheses of ST_a_ analogs for the development of vaccines or cancer probes [8].

**Figure 1:**
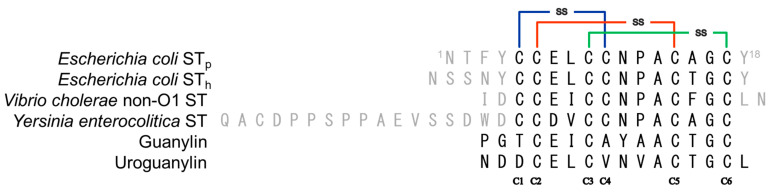
Amino acid sequences of the ST_a_-related peptides. Solid lines represent disulfide bonds.

ST_a_-like enterotoxins have been isolated from several bacterial species, such as *Vibrio cholerae* non-O1 and *Yersinia enterocolitica*, as shown in Figure 1 [9,10]. The toxic core of ST_a_ consists of thirteen amino acid residues and three intra-molecular disulfide bonds (C1-C4, C2-C5, and C3-C6 connectivity) [8,11,12]. The ST_a_ peptide is divided into ST_h_ and ST_p_ peptides, both of which have been purified from human and porcine strains of ETEC, respectively [13,14]. These peptides were chemically synthesized by the stepwise formation of the disulfide bonds and the products were characterized by X-ray crystallography [8,15]. The results indicated that the native form of the ST_p_ molecule consists of three β-turn moieties, with type I, type I, and type II β-turn structures in the N-terminal, central, and C-terminal regions, respectively [15]. It should also be noted that the central region (-Asn-Pro-Ala-Cys-) is responsible for receptor binding and is stabilized by an additional hydrogen bond between the carbonyl group of the sidechain of the Asn residue and the amide group of the Ala residue [15]. As a result, the ST_p_ molecule possesses a rigid structure that is maintained by disulfide bonds and β-turn structures. However, the restricted conformation also prevents the chemical synthesis of ST_a_ analogs for vaccine development because numerous disulfide isomers are produced during disulfide bond formation in the chemical synthesis. In addition, the N-terminal amino group of ST_a_ molecules are not very reactive due to steric hindrance, which makes further chemical modifications for conjugating with carrier proteins more difficult. Extending the N-terminal residues to introduce a more reactive amino group was also unsuccessful in producing the native form in high yields, since undesired disulfide isomers were produced during the chemical synthesis [16]. Thus, the locations on the molecule for introducing a functional group, such as an amino group, as a conjugation moiety were restricted. To solve these issues, we herein propose a strategy for constructing a form of ST_a_ that would be suitable for use as a vaccine or as a probe for detecting cancer cells.

For this purpose, the toxic core of the ST_a_ molecule was used to produce a modified form of ST_a_ that could be used as a candidate for a vaccine or cancer probe. The reduced form of ST_a_ spontaneously and exclusively folds into the native conformation with the correct disulfide pairings (C1-C4, C2-C5, and C3-C6) by air oxidation without need for any redox reagents, such as glutathione, indicating that the amino acid sequence of ST_a_ contains sufficient information to allow the molecule to fold only into the native conformation [8]. Thus, in this study, the Cys residues were essentially retained so as to maintain the correct conformation of the ST_a_ molecule by the disulfide bonds. Therefore, other positions in the ST_a_ peptide were explored for incorporating a Lys residue as a functional group for conjugation.

The amino acid residues (-Glu-Leu-) at the N-terminal region are located in the type I β-turn structure and it is known that they play a role in the toxic activity of the molecule. Replacing the -Glu-Leu- sequence with other amino acid residues, such as the -Asp-Val- sequence in *Yersinia* ST, results in a lower toxic activity that is a suitable candidate for a vaccine [17,18]. In addition, the replacement also allows cross reactions between guanylin and uroguanylin to be avoided since peptide hormones possess the same sequence at these positions, as shown in Figure 1 [3]. However, unfortunately, the incorporation of a Lys residue at these positions resulted in the formation of several disulfide isomers during the peptide synthesis [unpublished data], making it difficult to obtain the ST_a_ toxoid vaccine with the correct conformation.

The central moiety (-Asn-Pro-Ala-) participates in the type I β-turn structure and serves as a receptor binding site [19]. This region has a rigid structure and would be an ideal site as an antigen for an antibody to avoid the cross reactions with peptide hormones since guanylin and uroguanylin possess the -Tyr-Ala-Ala- and -Asn-Val-Ala- sequences at that site, respectively. Therefore, this region was excluded as a candidate for replacement.

Finally, in silico analyses were applied to the C-terminal region of the ST_a_ molecule. The C-terminal region (-Cys-Ala-Gly-Cys-) forms a type II β-turn structure [15]. It is known that a Gly residue at the third position is required for producing a type II β-turn structure [20]. The incorporation of an L-Lys residue to the third position in place of the Gly residue completely disrupts the formation of the correct conformation of the ST_a_ molecule, resulting in the production of numerous disulfide isomers. However, we hypothesized that the incorporation of a D-Lys residue into the third position in the turn structure would stabilize the type II β-turn structure. In addition, the N-terminal Cys residue was also replaced with a D-Cys residue to inhibit proteolytic degradation by amino peptidases in the intestine or blood. It has been reported that the incorporation of a D-Cys residue at the N-terminus allows the half-life time of ST_a_ to be efficiently extended to activate the receptor protein [18]. Thus, we designed the peptide sequence, D-Cys-Cys-Asp-Val-Cys-Cys-Asn-Pro-Ala-Cys-Ala-D-Lys-Cys as the vaccine candidate.

On the other hand, disulfide bond formation in the peptide synthesis was also considered to permit the correct conformation of the ST_a_ molecule to be obtained. To efficiently produce the target ST_a_ molecule with the native disulfide bonds, disulfide bonds were regioselectively formed by a stepwise method [8]. This method predominantly produces an ST_a_ topological isomer, in which the mainchain has a left-handed spiral structure, with the native disulfide bonds while the native form of the ST_a_ molecule has a right-handed spiral structure [21]. The toxic activity of the topological isomer was approximately 1/10 that of the native form of the corresponding ST_a_ peptide [8]. Thus, the topological isomer itself would also be useful as a vaccine candidate and it can be shifted to the native conformation of the ST_a_ molecule by a thiol reagent [21].

In this study, to establish the molecular basis of the ST_a_ molecule needed for use as a toxoid vaccine or cancer probe, we focused on the type II β-turn at the C-terminal region of the ST_a_ molecule and replaced the Gly residue with a D-Lys residue to produce a conjugation site. The designed ST_a_ peptide was also applied to specifically detect the GC-C protein, using Caco-2 cells or the GC-C protein that was recombinantly expressed on 293T cells.

## 2. Results and Discussion

### 2.1. Preparation of the D-Lys-Substituted ST_a_ Peptides

**Figure 2:**
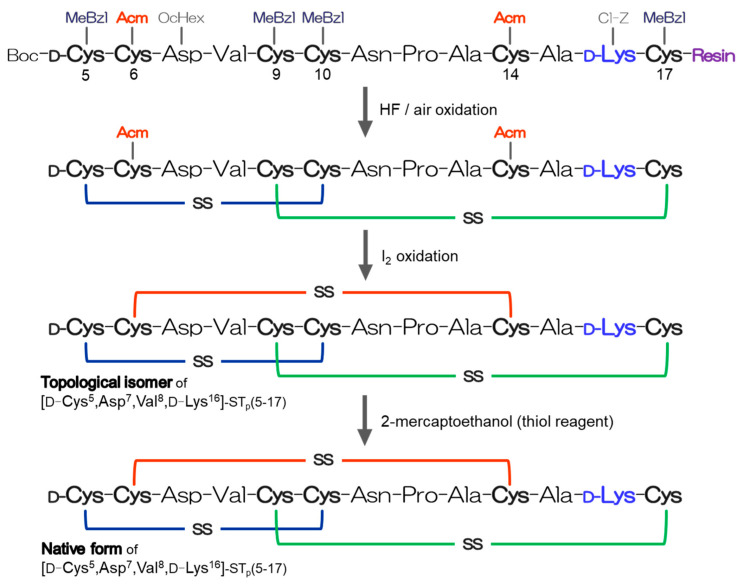
Scheme showing the synthesis of the [D-Cys^5^,Asp^7^,Val^8^,D-Lys^16^]-ST_p_(5-17) peptides with the correct disulfide bonds.

**Figure 3:**
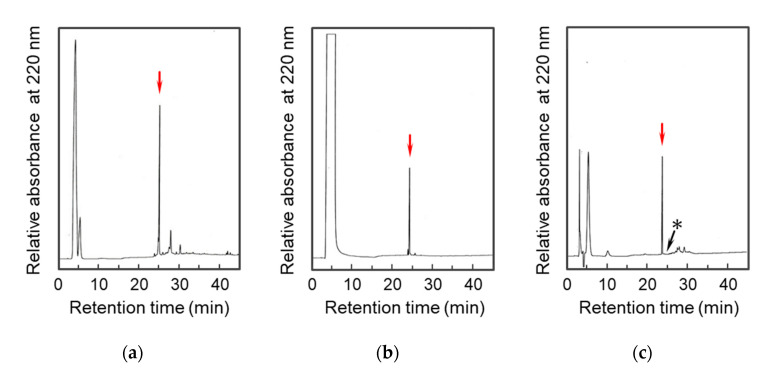
HPLC profiles of the preparation of the topological isomer and native form of [D-Cys^5^,Asp^7^,Val^8^,D-Lys^16^]-ST_p_(5-17) peptides. The air (**a**) and iodine (**b**) oxidations of the [D-Cys^5^,Cys^6^(Acm),Asp^7^,Val^8^,Cys^14^(Acm),D-Lys^16^]-ST_p_(5-17) with two disulfide bonds and the [D-Cys^5^,Asp^7^,Val^8^,D-Lys^16^]-ST_p_(5-17) peptide with three disulfide bonds, respectively. The thiol catalytic reaction (**c**) of the arrowed peak in (**b**). The target peptides are indicated by red arrows. The arrow with an asterisk indicates the position of the arrowed peak in (**b**).

To develop an ST_a_ vaccine, the candidate peptides, [D-Cys^5^,Asp^7^,Val^8^,D-Lys^16^]-ST_p_(5-17), were synthesized by a stepwise method for disulfide bond formation based on the scheme shown in Figure 2. After removing the protecting groups by a hydrogen fluoride (HF) treatment, the native disulfide bonds between D-Cys^5^ and Cys^10^ and between Cys^9^ and Cys^17^ were predominantly formed by air oxidation in the absence of redox reagents, as shown in Figure 2. Using our strategy, it was possible to obtain the correct/native disulfide pairings (the arrowed peak in Figure 3a) that stabilized the conformation of the precursor peptide with two disulfide bonds [8,12]. The disulfide bond between the Cys^6^ and Cys^14^ residues was then selectively formed by iodine oxidation with a recovery of ca. 89%, as shown in Figure 3b. It is important that only the topological ST_p_ isomer (the arrowed peak in Figure 3b) was produced using our strategy, as previously described [8,12]. The topological isomer has a left-handed spiral structure and generally showed a lower biological activity (approximately 1/10 that of the native ST_a_ peptide). This topological isomer was utilized as the ST_a_ vaccine candidate in this study.

To obtain the native form of the [D-Cys^5^,Asp^7^,Val^8^,D-Lys^16^]-ST_p_(5-17) peptide, the ST_p_ topological isomer was treated with 2-mercaptoethanol as a catalyst for isomerization. Only one peptide, which was assigned to the native form of the ST_p_ peptide as described below, was quantitatively produced in the reaction within a few minutes, as shown in Figure 3c. Importantly, no significant amounts of other disulfide isomers including the topological isomer were observed in the reaction, indicating that the reaction product is thermodynamically the most stable form.

As described in the introduction, it has been proposed that ST_a_ could be used for detecting cancer cells, such as Caco-2 or T84 cells since GC-C is predominantly located on cancer cells [6,7]. Interestingly, several studies have suggested that the activation of GC-C signaling in human colon cancer cells inhibits cell proliferation [22,23,24]. Thus, there are two possibilities for ST_a_ to detect cancer cells and be used as a therapeutic for treating metastatic colorectal cancer. To examine this issue further, an ST_a_ analog was labeled with fluorescein and was then used to detect colon cancer cells. For this purpose, [Mpr^5^,D-Lys^16^]-ST_p_(5-17) was also prepared to avoid side reactions between the N-terminal amino group and fluorescein isothiocyanate (FITC). Thus, the [Mpr^5^,D-Lys^16^]-ST_p_(5-17) peptide was also synthesized using our stepwise method for disulfide bond formation. The [Mpr^5^,Cys^6^(Acm),Cys^14^(Acm),D-Lys^16^]-ST_p_(5-17) peptide with two correct disulfide bonds was predominantly produced (the arrowed peak in Supplementary Materials Figure S1a) but, contrary to our expectations, the subsequent iodine oxidation yielded both the topological isomer and the native form of the [Mpr^5^,D-Lys^16^]-ST_p_(5-17) peptides (peaks 1 and 2 in Supplementary Materials Figure S1b). This result provided important structural information regarding the chemical synthesis of ST_a_ peptides, as we discussed in a previous report [8,12]. It is noteworthy that the steric hindrance at the N-terminal amino group regulates the construction of not only the topological isomer but also the native form of the [Mpr^5^,D-Lys^16^]-ST_p_(5-17) peptides. The conformations of the ST_a_ peptides were determined by CD spectroscopy. The results are discussed in the next section and peaks 1 and 2 in Supplementary Materials Figure S1b correspond to the topological isomer and native form of the [Mpr^5^,D-Lys^16^]-ST_p_(5-17) peptides, respectively, as described below. This result was also confirmed by the catalytic thiol reaction of those peptides. The peak 1 peptide changed to the peak 2 peptide in the disulfide exchange reaction, as shown in Supplementary Materials Figure S1c. It should also be noted that the peak 2 peptide had no effects in the reaction, as shown in Supplementary Materials Figure S1d, indicating that the peak 2 peptide in Supplementary Materials Figure S1b is the native form of the [Mpr^5^,D-Lys^16^]-ST_p_(5-17) peptide. Importantly, the results of the disulfide shuffling reaction using 2-mercaptoethanol also indicates that the final peptide (native form) can be obtained without regioselective disulfide bond formation when the D-Lys residue is incorporated into the third position of the type II β-turn in place of the Gly residue of the ST_a_ peptide.

The overall results obtained for the syntheses of the [D-Cys^5^,Asp^7^,Val^8^,D-Lys^16^]-ST_p_(5-17) and [Mpr^5^,D-Lys^16^]-ST_p_(5-17) peptides, D-Lys-substituted ST_a_ analogs indicate that these peptides spontaneously fold into the native conformation without other disulfide isomers being produced, even when the three intra-molecular disulfide bonds are simultaneously formed. This is a clear advantage in terms of the commercial production of ST_a_ analogs, especially for preparing a vaccine. It therefore appears that the D-Lys-substituted ST_a_ peptide can be conveniently synthesized and that it contains a functional group (-NH_2_) that allows for conjugation and chemical modification.

### 2.2. Conformational Analyses of the ST_p_ Peptides by Means of CD Spectroscopy

**Figure 4:**
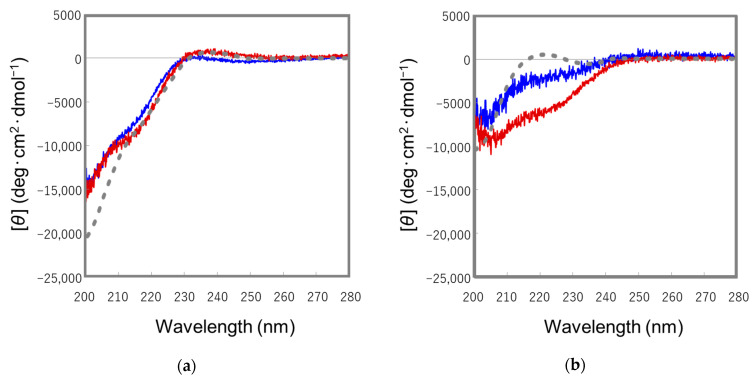
CD spectra of the native forms (**a**) and topological isomers (**b**) of the ST_p_ peptides. The red, blue, and gray colored lines represent the CD spectra of the [D-Cys^5^,Asp^7^,Val^8^,D-Lys^16^]-ST_p_(5-17), [Mpr^5^,D-Lys^16^]-ST_p_(5-17), and wild type ST_p_(5-17) peptides, respectively.

To determine the conformation of the synthetic peptides, CD measurements were carried out since the native form and topological isomer of ST_a_ peptides show characteristic CD spectra [25]. The CD spectra for the arrowed peak in Figure 3c and the peak 2 in Supplementary Materials Figure S1b are similar to that of the native form of the wild type ST_p_(5-17), indicating that both peptides correspond to the native forms of the designed ST_p_ peptides, as shown in Figure 4a. However, the arrowed peak in Figure 3b and peak 1 in the Supplementary Materials Figure S1b showed that the CD spectra were different from that of the topological isomer of wild type ST_p_(5-17), as shown in Figure 4b. These peptides are topological isomers of the designed ST_p_ peptides in our synthetic strategy. Therefore, to confirm the conformations of the topological isomers of the D-Lys-substituted ST_p_ peptides, we carried out the NMR measurements on the synthetic ST_p_ peptides.

### 2.3. The Solution Structure of the Topological Isomer of the [D-Cys^5^,Asp^7^,Val^8^,D-Lys^16^]-ST_p_(5-17) Peptide Determined by NMR Spectroscopy

To confirm the conformations of the synthetic ST_p_ peptides, the native form and topological isomer of the [D-Cys^5^,Asp^7^,Val^8^,D-Lys^16^]-ST_p_(5-17) peptides, NMR measurements were carried out. Two dimensional correlation spectroscopy (COSY) and nuclear Overhauser effect spectroscopy (NOESY) spectra were acquired and proton assignments were made to obtain distance constraints. Using combinations of these NMR spectra, almost of all of the proton signals could be assigned for both the native form and the topological isomer of the [D-Cys^5^,Asp^7^,Val^8^,D-Lys^16^]-ST_p_(5-17) peptides. The chemical shifts of the native form and topological isomer of the [D-Cys^5^,Asp^7^,Val^8^,D-Lys^16^]-ST_p_(5-17) peptides have been deposited in the Biological Magnetic Resonance Bank (BMRB) (https://bmrb.io/, accessed on 20 December 2022) under the accession code 51743 and 36529, respectively.

Over 100 distance constraints were obtained from the NOESY spectra of the native form of the [D-Cys^5^,Asp^7^,Val^8^,D-Lys^16^]-ST_p_(5-17) peptide. Unfortunately, almost all of distance constraints that were determined by NOESY were intra-residual or sequential (from residue *i* to *i* + 1) constraints, and the calculated structures did not converge precisely. The structure of the native form of the ST_p_ molecule was determined by an X-ray crystallography in a previous study [15]. The reported ST_p_ structure shows that all sidechains of the native form are exposed to solvent, suggesting that interactions between sidechains were minimal except for sequential residues. Furthermore, the CD spectrum of the native form of the [D-Cys^5^,Asp^7^,Val^8^,D-Lys^16^]-ST_p_(5-17) peptide was similar to that of the native form of the ST_p_ molecule [21,25]. These results suggest that the backbone structure of the native form of [D-Cys^5^,Asp^7^,Val^8^,D-Lys^16^]-ST_p_(5-17) is similar to that of the native ST_a_ molecule. In the case of the topological isomer, 179 distance constraints were obtained from NOESY spectra and were used in the structure calculation process. Using the CNS program, 500 structures were calculated and the 10 lowest total energy structures were selected, as shown in Supplementary Materials Figure S2. The overall average of the root mean square deviation (RMSD) value for the backbone heavy atoms and all heavy atoms, including the sidechains of the topological isomer, are summarized in Supplementary Materials Table S1. The superposition of structures showed that the calculated structures of the topological isomer converged well (Supplementary Materials Figure S2), suggesting that the structure of the topological isomer is tightly maintained by intra-molecular disulfide bonds. In the calculated structure of the topological isomer of the [D-Cys^5^,Asp^7^,Val^8^,D-Lys^16^]-ST_p_(5-17) molecule, as expected, the sidechain of the D-Lys residue protruded from the molecule.

**Figure 5:**
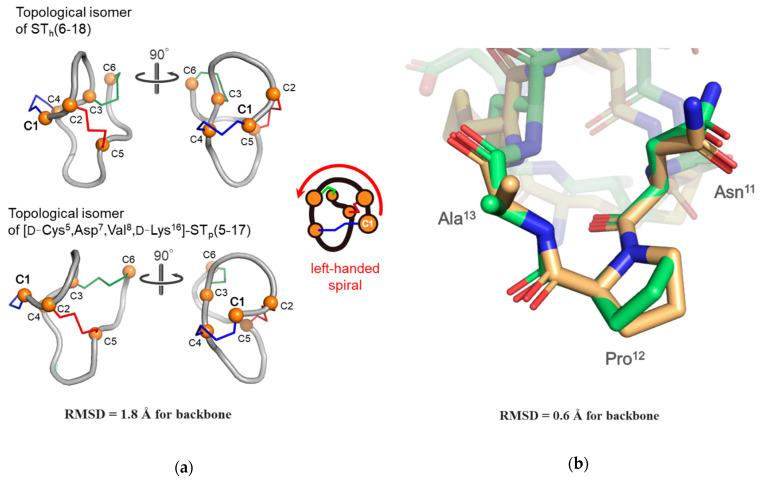
Structural comparisons between the topological isomers of wild type ST_h_(6-18) and [D-Cys^5^,Asp^7^,Val^8^,D-Lys^16^]-ST_p_(5-17) molecules (**a**). The whole structure of the topological isomers of the wild type ST_h_(6-18) (upper) and [D-Cys^5^,Asp^7^,Val^8^,D-Lys^16^]-ST_p_(5-17) (bottom) molecules are drawn as noodle models. The receptor binding site (**b**) of wild type ST_h_(6-18) (gold) and [D-Cys^5^,Asp^7^,Val^8^,D-Lys^16^]-ST_p_(5-17) (light green) molecules are depicted by stick models.

We previously determined the structure of the topological isomer of the wild type ST_h_(6-18), which adopted a left-handed spiral backbone structure and was distinctly different from the native conformation, which has a right-handed spiral backbone structure [21]. The topological isomer of the [D-Cys^5^,Asp^7^,Val^8^,D-Lys^16^]-ST_p_(5-17) peptide possessed a left-handed spiral backbone structure and showed a high structural similarity to that of the wild type ST_h_(6-18), as shown in Figure 5a. In the calculated structure, we confirmed that the structure of the receptor binding site (-Asn^11^-Pro^12^-Ala^13^-) in the [D-Cys^5^,Asp^7^,Val^8^,D-Lys^16^]-ST_p_(5-17) peptide was quite similar to that of the wild type ST_h_ peptide, as shown in Figure 5b [15]. However, the C-terminal region (-Cys^14^-Ala^15^-D-Lys^16^-Cys^17^) of the D-Lys-substituted ST_p_ molecule had a slightly different structure, compared to that of wild type ST_h_(6-18) molecule. The C-terminal region of the topological isomer of the wild type ST_h_(6-18) appears to be slightly flexible although the topological isomer of the D-Lys-substituted ST_p_ molecule forms a rigid type II β-turn structure at the C-terminal region. To estimate the conformation of the C-terminal region (-Cys^14^-Ala^15^-D-Lys^16^-Cys^17^) more precisely, we performed Ramachandran plot analyses for the [D-Cys^5^,Asp^7^,Val^8^,D-Lys^16^]-ST_p_(5-17) molecule. The Ramachandran plots showed that the φ and ψ values of the *i* + 1 (Ala^15^) and *i* + 2 (D-Lys^16^) residues in the topological isomer of the [D-Cys^5^,Asp^7^,Val^8^,D-Lys^16^]-ST_p_(5-17) and the native form of wild type ST_a_ molecules were located at the category of a type II β-turn structure [26]. However, those values for the topological isomer of wild type ST_h_(6-18) molecule could not be categorized as a type II β-turn structure (Supplementary Materials Figure S3), suggesting that this region has a flexible structure. These results suggest that the -Cys^14^-Ala^15^-D-Lys^16^-Cys^17^ region in the topological isomer of the [D-Cys^5^,Asp^7^,Val^8^,D-Lys^16^]-ST_p_(5-17) molecule definitely forms a type II β-turn structure as well as that of wild type ST_a_ molecule. The type II β-turn structure absolutely requires the presence of a Gly residue at the *i* + 2 position that corresponds to the position 16 or 17 in ST_p_(5-17) or ST_h_(6-18), respectively [27]. It has been reported that certain types of D-amino acid residues could stabilize β-turn structures [28]. In fact, the D-Lys^16^ residue was able to stabilize the local conformation of the C-terminal region of the [D-Cys^5^,Asp^7^,Val^8^,D-Lys^16^]-ST_p_(5-17) molecule. We therefore conclude that the substitution of the Gly residue to a D-Lys residue in the ST_a_ molecules results in the structural stabilization of the C-terminal region, as evidenced by the different CD spectra of the topological isomers of the D-Lys-substituted ST_p_ peptides from that of the topological isomer of wild type ST_a_ peptide.

In conclusion, the results obtained in this study indicate that the substitution of a D-Lys residue at the type II β-turn structure of the ST_a_ molecule results in the formation of an ST_a_ molecule with a much more rigid structure and also allows the correct conformation of the receptor binding site to be retained. Thus, we successfully introduced a functional group (-NH_2_) in an ST_a_ molecule for conjugation with other molecules, such as carrier proteins for vaccinations.

### 2.4. Detection of the GC-C Protein on Colon Cancer Cells

**Figure 6:**
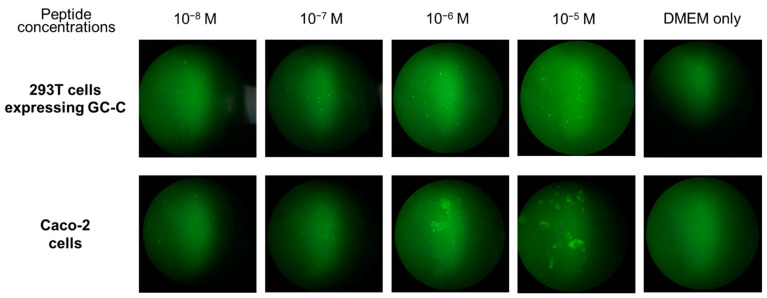
Fluorescence microscopy of 293T cells (**upper column**) expressing the recombinant GC-C protein and Caco-2 cells (**bottom column**). The native form of the [Mpr^5^,D-Lys^16^(FTC)]-ST_p_(5-17) was used in the binding assay.

To utilize the molecular basis of the ST_a_ peptide, the native form of the ST_p_ peptide was employed and its sensitivity for detection was examined using 293T cells expressing the recombinant GC-C protein [29]. For this purpose, the native form of the [Mpr^5^,D-Lys^16^]-ST_p_(5-17) peptide was prepared and treated with FITC to produce the [Mpr^5^,D-Lys^16^(FTC)]-ST_p_(5-17) peptide, as shown in Supplementary Materials Figure S4. The labeled ST_p_ peptide was confirmed by MALDI-TOF/MS and amino acid analyses. Only the 293T cells expressing the GC-C protein were observed by florescent microscopic analyses and the detection was concentration-dependent for the [Mpr^5^,D-Lys^16^(FTC)]-ST_p_(5-17) peptide, as shown in Figure 6. The labeled ST_p_ peptide was able to bind to the GC-C protein on 293T cells with a high sensitivity, even at a peptide concentration of 10^−8^ M, as shown in Figure 6. In addition, the labeled ST_p_ peptide could also be used to detect colon cancer cells, Caco-2 cells, by fluorescence analysis using a 10^−6^ M solution of the labeled ST_p_ peptide, as shown in Figure 6. The topological isomer of the labeled ST_p_ peptide was also examined for the detection of cancer cells and showed a superior ability to detect the cells as well as the native form of the ST_p_ peptide (data not shown). Thus, the designed peptide showed a superior ability not only as a potential ST_a_ vaccine candidate but also as a probe for detecting cancer cells expressing the GC-C protein.

### 2.5. Receptor Binding Activity of the Synthetic ST_p_ Peptide

**Figure 7:**
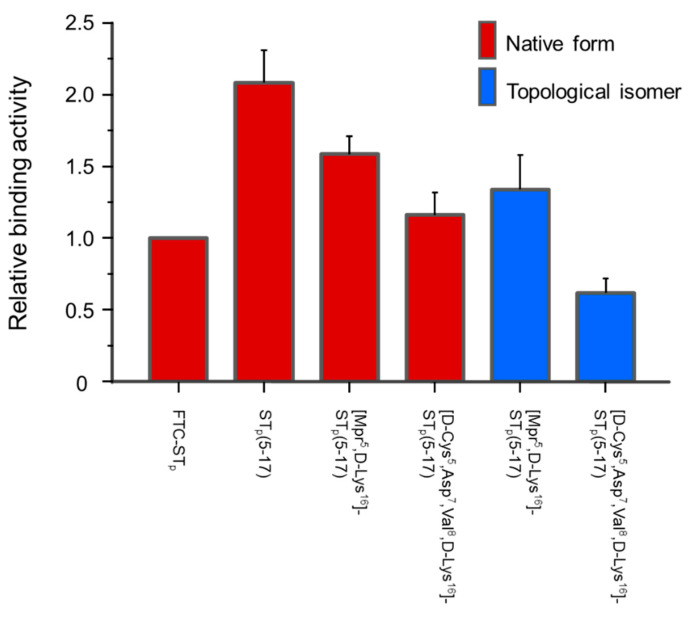
Competitive binding assay of the synthetic ST_p_ peptides. The binding activity of the FTC-ST_p_ peptide was normalized to a binding activity of 1.0.

To confirm the toxic activities of the ST_p_ peptides, GC-C binding assays were performed using the recombinant GC-C protein on 293T cells, as shown in Figure 7. It has been reported that the replacement of the N-terminal L-Cys residue with a D-Cys or an Mpr residue do not significantly affect the toxic activity of ST_p_ using a suckling mouse assay, although the receptor binding activities were not determined precisely [15,18]. The toxicities of the native form and the topological isomer of the [Mpr^5^,D-Lys^16^]-ST_p_(5-17) and the [D-Cys^5^,Asp^7^,Val^8^,D-Lys^16^]-ST_p_(5-17) peptides were compatible with those of the [Mpr^5^]-ST_p_(5-17), [D-Cys^5^]-ST_p_(5-17), and *Yersinia* ST peptides [17,18], indicating that the conformations responsible for the toxic activity are largely retained in the designed ST_p_ molecules. As expected, the replacement of the -Glu-Leu- to -Asp-Val- sequences in the [D-Lys^16^]-ST_p_(5-17) peptide decreased the GC-C binding activity [17]. In addition, the topological isomers of all the peptides showed lower toxic activities than that of each individual ST_p_ peptide. Thus, the results of the GC-C binding assay were also consistent with previously reported results [8], indicating that the incorporation of the D-Lys residue into the C-terminal region is suitable for producing ST_p_ molecules with a rigid structure. It has been reported that the topological isomer and the -Asp-Val- sequence reduced the toxic activities of the peptide to approximately 1/10 and 1/20 (MED: 6.9 nmol, 9.7 nmol) of that of the native ST_p_ or ST_h_ peptides (MED: 0.4 nmol), respectively [8,12,17]. Therefore, the results obtained in this study suggest that the toxic activity of the [D-Cys^5^,Asp^7^,Val^8^,D-Lys^16^]-ST_p_(5-17) is approximately 1/200 lower than that of the native ST_p_ peptide with the correct conformation at the receptor binding site, as evidenced by NMR measurements. Thus, the designed ST_p_ peptide provides a ST_a_ toxic moiety which is an appropriate site to allow expected antibodies for therapy to be produced without the cross reaction to the peptide hormones, such as guanylin and uroguanylin.

### 2.6. Design of ST_a_ Analogs Using the [D-Lys^16^]-ST_p_(5-17) Peptide as a Molecular Basis for Vaccine Development

In this study, a D-Lys residue was incorporated into the ST_p_ molecule to provide the functional amino group for a conjugation site. The results obtained in this study clearly indicate that the D-Lys residue stabilized the type II β-turn structure in the ST_a_ molecule without affecting toxic activity. In addition, an ST_a_ peptide vaccine with the native type of disulfide bonds can also be easily produced without the need for a specific intermediate method, such as regioselective disulfide formation, thus greatly reducing the cost for the vaccine preparation.

The focus on the [Ser^9^,Thr^14^]-ST_h_ peptide, which was originally prepared by recombinant methodology, was on the development of an ST_a_ vaccine [30]. The reported [Ser^9^,Thr^14^]-ST_h_ peptide has a reduced toxicity in excess of 100-fold for each mutation and is recognized by several antibodies, indicating that it is an appropriate candidate for vaccine development. However, we believe that the [Leu^13^]-ST_p_(5-17) (MED, >6600 pmol) is a much more suitable candidate for use in a vaccine since the ST_a_ molecule can be produced in high yield and is quite stable [19]. In addition, [Ser^9^]-ST_h_(6-19) with the native disulfide bonds still showed a moderate toxicity (MED, 14 pmol) in our experiments using the chemically synthesized [Ser^9^]-ST_h_(6-19) peptide [17]. It should also be noted that non-native disulfide isomers are often recognized by the ST_a_ antibody. It therefore appears that the [D-Cys^5^,Asp^7^,Val^8^,Leu^13^,D-Lys^16^]-ST_p_(5-17) peptide represents a superior antigen when a nontoxic form of the ST_a_ peptide is absolutely required. However, the [Mpr^5^,D-Lys^16^]-ST_p_(5-17) and [D-Cys^5^,Asp^7^,Val^8^,D-Lys^16^]-ST_p_(5-17) peptides, synthesized in this study, are suitable for use as probes for the detection of cancer, since the [D-Cys^5^,Asp^7^,Val^8^,Leu^13^,D-Lys^16^]-ST_p_(5-17) peptide lacks the receptor binding activity.

## 3. Materials and Methods

### 3.1. Materials

All chemicals and solvents were reagent grade unless otherwise described. The Boc amino acid derivatives were purchased from the Peptide Institute Inc. (Osaka, Japan).

### 3.2. Peptide Synthesis

The peptides were manually synthesized by the solid-phase method, as described previously [8,12,31]. The disulfide bonds were regioselectively formed by a stepwise method using air and iodine oxidation, as shown in Figure 2. To detect cancer cells, the [Mpr^5^,D-Lys^16^]-ST_p_(5-17) peptide (5 nmol) in 1% NaHCO_3_ (25 μL, pH 8.8) was typically treated with 1 mM fluorescein isothiocyanate (FITC) at room temperature for 16 h in order to synthesize the [Mpr^5^,D-Lys^16^(FTC)]-ST_p_(5-17) peptide. All peptide analogs prepared in this study were purified and identified by means of reversed-phase HPLC (RP-HPLC) and matrix assisted laser desorption/ionization time of flight mass spectrometry (MALDI-TOF/MS).

### 3.3. Reversed-Phase High Performance Liquid Chromatography (RP-HPLC)

The HPLC apparatus was comprised of a HITACHI ELITE LaChrom system (L2130) equipped with a Hitachi L-3000 detector and a D-2500 chromato-integrator. Peptides were purified by RP-HPLC using a Cosmosil 5C_18_-AR-II column (4.6 × 150 mm, Nacalai tesque Inc., Kyoto, Japan). The peptides were separated by a linear gradient of CH_3_CN in 0.05% TFA increasing at a rate of 1%/min from solvent A (0.05% TFA/H_2_O) to solvent B (0.05% TFA/CH_3_CN) at a flow rate of 1 mL/min [8,21].

### 3.4. Matrix Assisted Laser Desorption/Ionization Time of Flight Mass Spectrometry (MALDI-TOF/MS)

The molecular masses of the peptides were determined by means of an AXIMA confidence spectrometer (SHIMADZU Co., Kyoto, Japan) in the positive ion mode [21]. Mass spectrometric analyses of peptides were carried out in the reflector mode using α-cyano-4-hydroxycinnamic acid (Tokyo Chemical Industry Co., Ltd., Tokyo, Japan) as a matrix. In a typical run, the lyophilized peptide (ca. 0.1 nmol) was dissolved in 0.05% TFA aq/50% CH_3_CN (1 μL), mixed with 1 μL of a matrix solution (10 mg/mL), and air-dried on the sample plate for use in MALDI-TOF/MS.

### 3.5. Circular Dichroism (CD) Measurement

The peptide concentrations were determined by amino acid analyses [21]. The peptides (0.1 mg/mL) were dissolved in 50 mM sodium phosphate buffer (pH 7.0). CD spectra were recorded on a JASCO J-820 spectropolarimeter (Tokyo, Japan) at room temperature.

### 3.6. NMR Measurements and Structure Calculations

All NMR experiments were carried out at 25 °C on a JNM-ECA800 spectrometer (JEOL RESONANCE Inc., Tokyo, Japan). The peptide samples for NMR experiments were dissolved to a final peptide concentration of approximately 3 mM in 20 mM sodium phosphate buffer prepared with D_2_O or a 90% H_2_O/10% D_2_O mixture at pH 6.5 and transferred to a 5 mm NMR microtube (Shigemi, Tokyo, Japan) [21]. A series of 1D and 2D spectra, including ^1^H, DQF-COSY and ^1^H-^1^H NOESY, were obtained and were used to assign each proton signal of the amino acid residues. The chemical shifts of the native form and topological isomer of [D-Cys^5^,Asp^7^,Val^8^,D-Lys^16^]-ST_p_(5-17) have been deposited in the Biological Magnetic Resonance Bank (BMRB) (https://bmrb.io/, accessed on 20 December 2022) under the accession code 51743 and 36529, respectively. The NOE distance constraints for peptides were derived from ^1^H-^1^H NOESY spectra with mixing times of 200 msec. All structure calculations were performed with the CNS program [32]. Structure optimization and energy minimization were achieved using a simulated annealing algorithm. The final 10 lowest energy structures were analyzed using the MOLMOL [33] and PROCHECK [34] programs. Structural statistics for the 10 structures are included in Supplementary Materials Table S1. Graphical representations were prepared using PyMOL (www.pymol.org, accessed on 20 December 2022). The structures of the topological isomer of [D-Cys^5^,Asp^7^,Val^8^,D-Lys^16^]-ST_p_(5-17) have been deposited in the Protein Data Bank (PDB) (http://www.rcsb.org/pdb/, accessed on 20 December 2022) under the accession code 8HR3.

### 3.7. Detection of the ST_a_ Receptor Using the Fluorescein Labeled ST_a_ Peptide

The recombinant ST_a_ receptor was expressed by 293T cells, as previously described with minor modifications [29]. Briefly, mammalian 293T cells were grown and maintained in DMEM with 10% fetal bovine serum (FBS) in a 60 mm diameter or 24-well plates [35]. Receptor expression was performed using TransIT-293 Transfection Reagent (Mirus, Wisconsin, USA) according to the protocol provided by the manufacture. The transfected cells were incubated at 37 °C for 72 h in a 5% CO_2_ incubator.

The GC-C expressing 293T or Caco-2 cells on 24-well plates were washed with PBS (300 μL) and then were treated with the FTC-ST_p_ peptide, [Mpr^5^,D-Lys^16^(FTC)]-ST_p_(5-17), in DMEM (500 μL) without FBS at room temperature for 30 min. After washing with PBS (500 μL), DMEM (500 μL) was added to each well and the resulting cells were observed with a fluorescein microscope using a FITC filter set.

### 3.8. Competitive Binding Assay Using the Recombinant GC-C on 293T Cells

The 293T cells expressing GC-C on a 10 cm dish were harvested with PBS (5 mL) and centrifuged at 3500 rpm for 10 min at 4 °C. The cells were suspended with DMEM (5 mL) and then divided into small portions (182 μL). The aliquots were again centrifuged at 3500 rpm for 10 min at 4 °C and re-suspended/mixed with the FTC-ST_p_ peptide (10^−5^ M) solution in DMEM (200 μL) containing 10^−5^ M ST_p_ analogs at room temperature for 30 min. The reaction mixtures were centrifuged at 3500 rpm for 10 min at 4 °C, resuspended with DMEM (500 μL), transferred to a 24-well plate, incubated for 1 h at 37 °C in a 5% CO_2_ incubator, and observed by fluorescein microscopy using a FITC filter set. The fluorescein densities of each cells were analyzed by the software Image J. Binding assays were conducted in duplicate.

## 4. Conclusions

Based on the findings obtained in this study, we propose a convenient method for the synthesis of candidate ST_a_ peptides as potential ST_a_ vaccines and for development of a cancer detection probe. The method provides the ST_a_ peptide with the desired conformation including the correct disulfide pairings as antigens and as probes for binding to the GC-C receptor protein. Vaccination treatments using the [D-Cys^5^,Asp^7^,Val^8^,D-Lys^16^]-ST_p_(5-17) and the proposed [D-Cys^5^,Asp^7^,Val^8^, Leu^13^,D-Lys^16^]-ST_p_(5-17) peptides are currently in progress for use in developing countries. In addition, the ST_a_ analog synthesized by our method is able to detect cancer cells and can be used for therapeutic treatments.

## Data Availability

Not applicable.

## Supplementary Materials

The following supporting information can be downloaded at: https://www.mdpi.com/article/10.3390/molecules28031128/s1, Table S1: Data collection and refinement statistics; Figure S1: HPLC profiles of the preparation of the topological isomer and the native form of the [Mpr^5^,D-Lys^16^]-ST_p_(5-17) peptides; Figure S2: Superposition of the calculated NMR structures of the topological isomer of the [D-Cys^5^,Asp^7^,Val^8^,D-Lys^16^]-ST_p_(5-17) molecule; Figure S3: Ramachandran plots of the φ and ψ values of the amino acid residues which participate in the C-terminal region of the ST_p_ molecules; Figure S4: HPLC profiles of the FITC (a) and reaction mixtures (b) with the [Mpr^5^,D-Lys^16^]-ST_p_(5-17) peptide.

## Abbreviations

Acm	acetamidomethyl
Boc	*t*-butyloxycarbonyl
CD	circular dichroism
Cl-Z	2-chlorobenzyloxycarbonyl
COSY	two dimensional correlation spectroscopy
DMEM	Dulbecco’s modified eagle medium
DQF	double quantum filtered
ETEC	Enterotoxigenic *Escherichia coli*
FBS	fetal bovine serum
FITC	fluorescein isothiocyanate
FTC	fluorescein thiocarbonate
GC-C	guanylyl cyclase C
MALDI-TOF/MS	matrix-assisted laser desorption/ionization time of flight mass spectrometry
MeBzl	methyl benzyl
MED	minimum effective dose
Mpr	mercaptopropionyl
NOE	nuclear Overhauser effect
NOESY	nuclear Overhauser effect spectroscopy
OcHex	O-cyclohexyl
PBS	phosphate buffered saline
RMSD	root mean square deviation
RP-HPLC	reversed-phase high performance liquid chromatography
ST_a_	heat-stable enterotoxin
ST_h_	heat-stable enterotoxin derived from an *E. coli* strain from human
ST_p_	heat-stable enterotoxin derived from an *E. coli* strain from porcine
TFA	trifluoroacetic acid
