# Supplementary material for: The Molecular Basis of Heat-Stable Enterotoxin for Vaccine Development and Cancer Cell Detection
Source: Molecules. 2023 Jan 23;28(3):1128. doi: 10.3390/molecules28031128 (PMC9920858; doi:10.3390/molecules28031128)
Supplement: Supplementary file 1 [file molecules-28-01128-s001.zip › molecules-2153258-supplementary.pdf]

Supplementary Materials

Table S1. Data collection and refinement statistics.

No. of restraints	**[D-Cys[5],Asp[7],Val[8],D-Lys[16]]-ST_P(5-17) topological isomer**
All	179
NOE distance restraints	176
Intra-residual	95
Sequential	43
Medium range	20
Long range	18
Disulfide bond	3
Deviations from idealized covalent geometry	
Bonds (Å)	0.0628 ± 4.2e-5
Angles (º)	0.516 ± 1.9e-3
Impropers (º)	0.291 ± 4.5e-3
Mean coordinate RMSD from mean structure[a]	
Backbone heavy atoms	0.12 ± 0.05 Å
All heavy atoms	0.44 ± 0.14 Å

[a] Root mean square deviation (RMSD) was calculated using 10 possible structures.

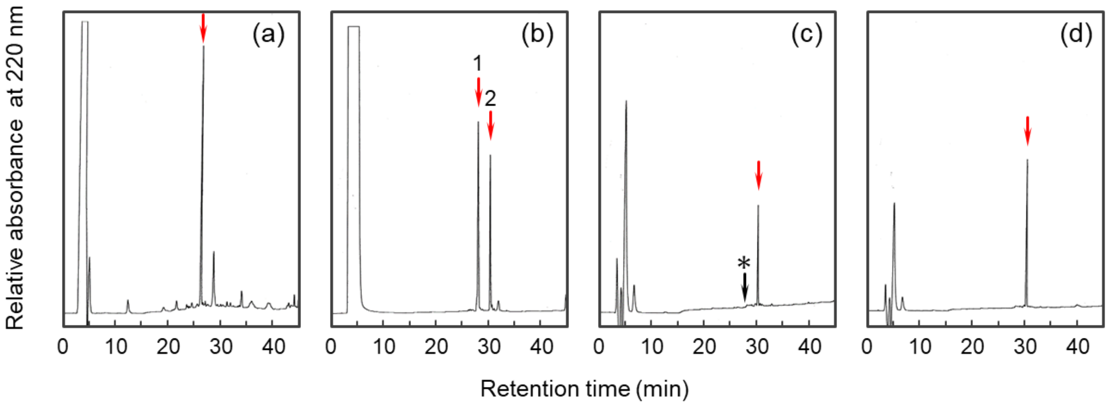

Figure S1. HPLC profiles of the preparation of the topological isomer and the native form of the [Mpr[5],D-Lys[16]]-ST_P(5-17) peptides. The air (a) and iodine (b) oxidations of the [Mpr[5],Cys[6](Acm),Cys[14](Acm),D-Lys[16]]-ST_P(5-17) with two disulfide bonds and the [Mpr[5],D-Lys[16]]-ST_P(5-17) peptide with three disulfide bonds, respectively. The thiol catalytic reaction (c and d) of the peaks 1 and 2 in (b), respectively. The arrow with an asterisk in (c) indicates the position of the peak 1 in (b). The target peptides were indicated by red arrows.

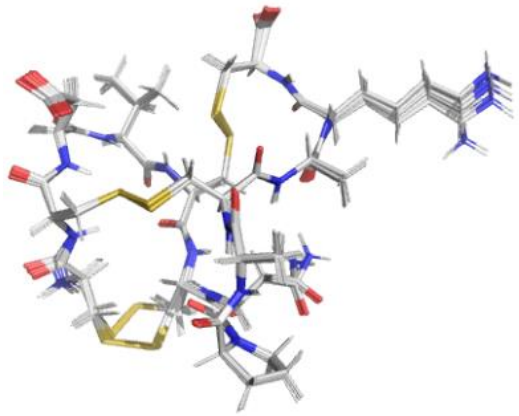

Figure S2. Superposition of the calculated NMR structures of the topological isomer of the [D-Cys^5,Asp^7,Val^8,D-Lys^{16}]-ST$_P$(5-17) molecule.

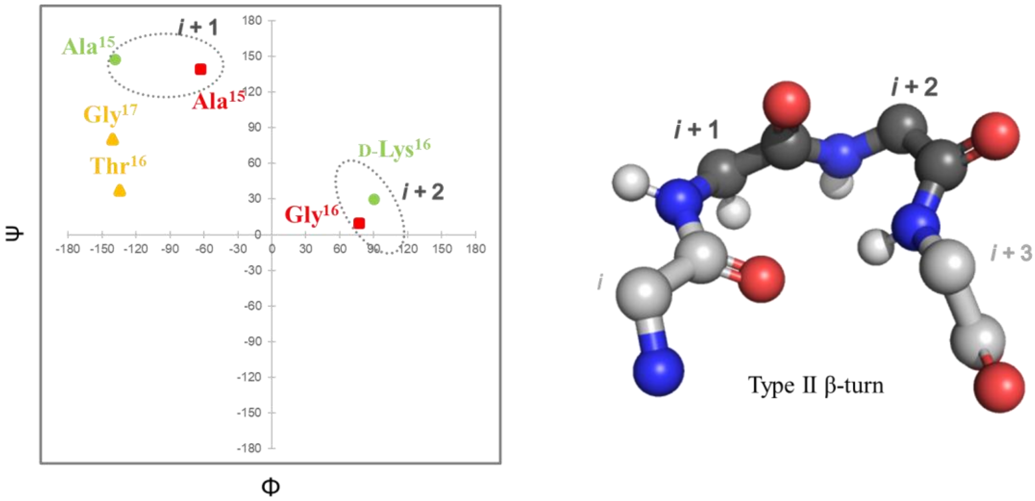

Figure S3. Ramachandran plots of the φ and ψ values of the amino acid residues which participate in the C-terminal region of the ST_P molecules. Red, orange, and light green colors indicate the values for the native form (PDB ID: 1ETN) and the topological isomer (PDB ID: 7CSS) of wild type ST_a molecules and the topological isomer (PDB ID: 8HR3) of the [D-Cys^5,Asp^7,Val^8,D-Lys^{16}]-ST_P(5-17) molecule, respectively.

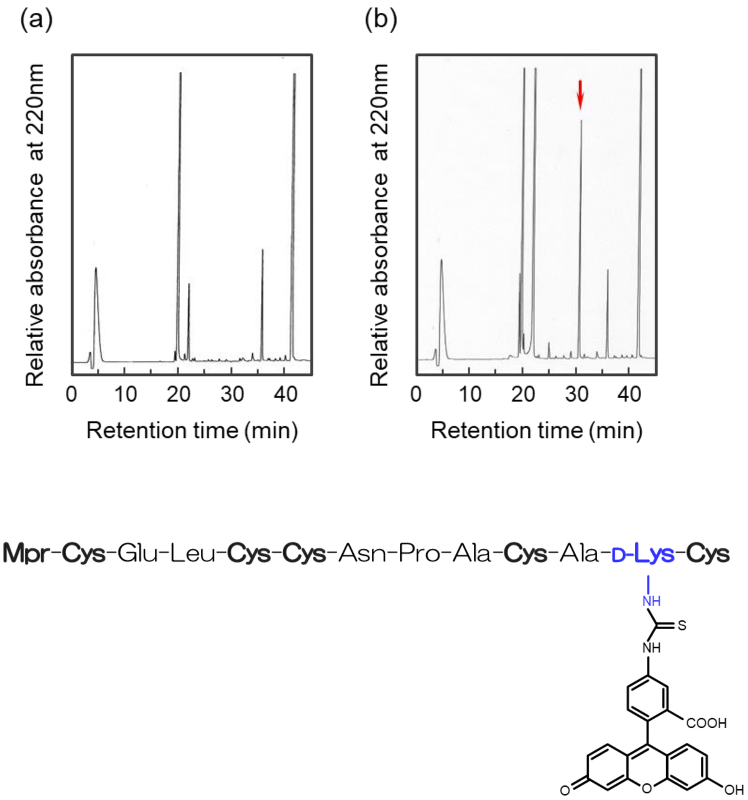

Figure S4. HPLC profiles of the FITC (a) and reaction mixtures (b) with the [Mpr^5,D-Lys^{16}]-ST$_P$(5-17) peptide. The arrowed peak in (b) corresponds to [Mpr^5,D-Lys^{16}(FTC)]-ST$_P$(5-17).
